# Talking about end of life in general palliative care – what’s going on? A qualitative study on end-of-life conversations in an acute care hospital in Denmark

**DOI:** 10.1186/s12904-019-0448-z

**Published:** 2019-07-25

**Authors:** Heidi Bergenholtz, Helle Ussing Timm, Malene Missel

**Affiliations:** 1 Holbaek Hospital, Smedelundsgade 60, 4300 Holbæk, Region Zealand Denmark; 2 REHPA, Knowledge Centre for Rehabilitation and Palliative Care, National Institute of Public Health, University of Southern Denmark, Vestergade 17, 5800 Nyborg, Denmark; 3 Rigshospitalet, Blegdamsvej 9, 2000 Copenhagen, Denmark

**Keywords:** Palliative care, Generalist palliative care, Hospital, End-of-life, Conversation, Interpretive description, Ethnography

## Abstract

**Background:**

End-of-life (EOL) conversations in hospital should serve to give patients the opportunity to consider future treatment options and help them clarify their values and wishes before it becomes relevant to make decisions about treatment. However, it is known that EOL conversations are not performed systematically in hospital. This may mean that patients and their relatives do not address EOL issues. There is a lack of knowledge about who is responsible for conducting these conversations, and when and under what circumstances they are conducted. The aim of this study was to explore the existing practices regarding EOL conversations in an acute care hospital setting.

**Methods:**

The design was Interpretive Description and the methods for the data collection included: 1. Participatory observational studies in a pulmonary medical and surgical ward (a total of 66 h); 2. Four focus group interviews with healthcare professionals (*n* = 14) from the wards. The analysis followed Spradley’s ethnosemantic analysis.

**Results:**

The results revealed three cultural categories related to: 1. The physical and organizational setting; 2. The timing of EOL conversations and competencies and roles in addressing EOL issues and 3. Topics addressed in EOL conversations. The EOL conversations were part of daily clinical practice, but there was a lack of competencies, roles were unclear and the physical and organizational environment was not conducive to the conversations. The topics of the EOL conversations revolved around a “here-and-now” status of the patient’s disease progression and decisions about the level of treatment. To a lesser extent, the conversations included the patient’s and relatives’ thoughts and wishes concerning EOL, which allowed long-term care planning.

**Conclusion:**

This study demonstrates that there are several barriers to talking about EOL in an acute care hospital setting, and future strategies must address an overall approach. In order to provide patients and their relatives with better opportunities to express their EOL wishes, there is a need for clearer roles and guidelines in an interdisciplinary approach to EOL conversations, alongside improved staff competencies and changes to the organizational and physical environment.

## Background

The Danish healthcare system does not have a tradition of talking with patients and their relatives about end of life (EOL) issues in a systematic manner [1]. This may mean that healthcare professionals (HPs) have no knowledge of patients’ wishes and priorities at the EOL; patients and their relatives may not have had the opportunity to decide whether they desire treatment, for how long they would like the treatment to continue, or how they plan to spend their remaining time.

EOL conversation is a common concept for conversations between professionals, patients, and relatives concerning wishes and decisions about the end of life. The aim of these conversations is to give the patient and their relatives the opportunity to decide on their EOL wishes in advance. Topics include life-prolonging treatment, as well as prognosis and preferences in EOL care (place of death and how to spend their remaining time) [2]. Thus, end of life is talked about before the actual end of the patient’s life [3]. In Denmark EOL conversations are addressed in the general recommendations for palliative care (PC) [4], and the Ethical Council also gives recommendations regarding EOL conversations [2]. These recommendations encourage HPs to address EOL issues at the earliest possible stage in a life-threatening disease trajectory in order to plan future care and treatment. However, they are only recommendations and it is left to the individual institution and the HPs to decide how and when such conversations are conducted and by whom. There are no formal guidelines regarding the competencies or educational level required for conducting these conversations. However, by law it is stated that only doctors may make decisions and document the level of treatment [5].

Talking about EOL has become an issue around the world, and many organizations have been established with the particular purpose of guiding people in talking about death and dying [6–8]. This has come about in order to break down taboos concerning death and dying. A systematized way to address EOL matters has been demonstrated in the Advanced Care Planning (ACP) [9, 10], which is a well-known approach to talking about EOL, also for chronic diseases [11, 12] and in hospital settings [13]. However, this approach has not been adopted systematically in hospitals, indicating that there are barriers to its implementation [14].

EOL conversations often take place in hospital, as many patients have several hospitalizations and contacts with the health system during their final years of life [15], and in Denmark 44% of all deaths occur in hospital [16]. EOL conversations can be characterized as part of PC, which aims to relieve suffering in patients with a life-threatening disease, regardless of diagnosis [4]. A crucial point in a patient trajectory is often the point when cure is no longer an option and further treatment is about the relief of symptoms and a focus on the quality of life.

Talking about EOL matters has been shown to be associated with reduced costs and better quality of care in the final weeks of life [17, 18]. Doctors’ (as well as nurses’) competence to talk about death and dying is, therefore, an essential component of PC. When patients are not offered a conversation, it is said to be due to the fact that doctors experience uncertainty in talking to patients about such sensitive matters [19, 20].

The target group of patients who may be in need of EOL conversations is still unclear, and one of the main challenges described at the generalist PC level^1^ is early identification of patients with PC needs [21], who may need these conversations. This lack of identification is due in part to the prognostic difficulty of identifying PC needs in patients with chronic diseases [22–24], and in part to the hospital’s primary focus on survival and life-prolonging treatment [25–27]. This gives rise to several barriers in the initiation of EOL conversations [17], especially for patients with chronic disorders [28], despite the fact that patients are often able to formulate their EOL wishes [29].

Although there is evidence for the necessity to address EOL issues with patients suffering from life-threatening illnesses, it remains to be illuminated how – or indeed, whether – this actually takes place in an acute care setting in hospital. What are the actual topics addressed? When, and under what circumstances, are the conversations taking place?

The aim of this study is to explore existing practices regarding EOL conversations in an acute care hospital setting. This is in order to gain knowledge on how to provide better opportunities for patients and their relatives to express EOL wishes.

## Methods

### Design

The study was a single-center descriptive qualitative study, using Interpretive Description [30] for the design, and participant observation [31] and focus group interviews [32, 33] as methods for data collection.

The overall methodology was Interpretive Description (ID) developed by Thorne [30]. Since the study has been developed from a clinical problem and was conducted in a clinical setting, ID offers a methodology with a nuanced understanding of the human experience and produces knowledge in a contextual understanding. ID is an inductive approach which draws on elements from both phenomenology, grounded theory and ethnography [30]. However, ID differs from other methodologies as it renounces the use of formalized techniques and procedures as the ultimate standards for research, striving instead to generate usable knowledge which can be applied in clinical practice. This means that ID design can generate knowledge that can contribute to action and inform nursing practice without being restrained by an external theoretical framework [34, 35].

However, the authors found it helpful to be guided by Spradley’s ethnographic method, which provides an opportunity to grasp the insiders’ point of view in order to better understand the context [31]. As the ID offers little guidance on the actual conduct of the study or the analysis, Spradley’s method was used. It should be noted that Spradley’s method does not differ substantially from the ID, but is more explicit. ID also recommends that the data be gathered and analyzed concurrently, allowing the preliminary data analysis to guide the subsequent data collection, such as the focus group interviews. This meant that the preliminary results from the field study were further discussed during the focus group interview, allowing the respondents to further explore their experiences with EOL conversations.

The researcher (first author) who conducted the field study and was the focus group moderator was also trained as a nurse with experience in both medical and surgical specialties.

### Clinical setting

The study took place in an acute hospital in Denmark with 338 beds, where 1,298 deaths occurred in 2017. A total of 1,700 people are employed at the hospital.

The study focuses on two selected inpatient wards: the pulmonary medical ward and the acute surgical ward. The wards were selected because of their interest in participating in the study and because they had patients with differing diagnoses. The pulmonary medical ward included patients with malignant and non-malignant diagnoses, whereas the acute surgical ward primarily included patients with malignancies receiving PC. The departments were similar with regard to the numbers of beds, nurses and doctors, as well as with regard to how the daily practice was organized. The physical environment was similar with rooms containing one to four beds.

### Material

#### Participant observation

During January and February 2018, two participant observation studies (referred to as one field study) [31] were conducted in the pulmonary medical and surgical wards by the first author. The researcher spent 2 weeks in each ward. A total of 66 h of conversations were observed, mainly during the day shift, when most of these conversations took place. The observations ranged from passive to moderate participation [31], as the researcher did not participate directly in the healthcare work but followed the HPs (both doctors and nurses) in their daily practice, including their conversations with patients about EOL issues.

Both formal and informal conversations were observed. The formal conversations were planned by the HPs and often took place during rounds where both a doctor and a nurse were present. A total of 27 formal conversations were followed during ward rounds (20 from the pulmonary ward and seven from the surgical ward), as well as 12 informal conversations with patients, relatives and HPs. The informal conversations sometimes took place after the formal conversations if the patients, relatives or HPs had some follow-up information. All of the conversations observed in the surgical ward were with cancer patients. In the pulmonary ward, conversations were held with patients with both malignant and non-malignant diagnoses.

During the field study, the researcher wrote field notes, in the form of brief sentences in a notebook, to keep track of the physical space and the words used in the conversations. Immediately after the observations, these notes were used to write expanded accounts [33], which formed the basis for the analysis. In total, 48 pages of field notes were compiled.

#### Focus group interviews

Following the field study, four focus group interviews with HPs (nurses and doctors) were conducted in order to clarify and elaborate on the observations made in the field study. The first author was the moderator for the interviews and the third author was an observer. A semi-structured interview guide was used and the topics included: *Experiences with EOL conversations, roles (own and others) in EOL conversations, the timing of EOL conversations, competencies in participating/initiating/conducting conversations, reflections on any experiences of discomfort, reflections on own mortality and topics addressed in EOL conversations.*

A total of 14 respondents participated (Table 1) in four interviews with three to four respondents in each. Six doctors and eight nurses participated (the latter being either nurses or social and healthcare assistants); the age range was 25–64 years; one was male; some were experienced and others were newly qualified. The respondents were chosen by the head nurse on each ward using purposive sampling [30] (the selection of those who had shown interest in the topic and wished to participate). The respondents were invited by the first author by e-mail, informing them of the purpose of the study, assuring their anonymity and stressing that participation was voluntary. All agreed to participate. The interviews were conducted in the hospital and were audio-recorded and transcribed verbatim. They lasted between 32 and 55 min.

**Table 1:** Respondents in the focus group interviews

Respondent	Profession	Sex F/M	Age	Seniority/year	Ward	Participating in focus group 1–4
1	Social and healthcare *assistant*	F	50–59	3	Pulmonary	1
2	Nurse	F	30–39	12	Pulmonary	1
3	Social and healthcare assistant	F	20–29	1	Pulmonary	1
4	Nurse	F	40–49	1	Pulmonary	1
5	Chief physician	F	60–69	40	Pulmonary	2
6	Physician	F	30–39	7	Pulmonary	2
7	Chief physician	F	30–39	10	Pulmonary	2
8	Chief physician	F	50–59	16	Surgical	3
9	Chief physician	F	50–59	10	Surgical	3
10	Chief physician	M	60–69	10	Surgical	3
11	Social and healthcare assistant	F	40–49	2 ½	Surgical	4
12	Nurse	F	50–59	2	Surgical	4
13	Nurse	F	20–29	½	Surgical	4
14	Nurse	F	20–29	1 ½	Surgical	4

### Analysis

The analysis followed Spradley’s ethnosemantic analysis [31, 36], which is a includes a domain, taxonomic and componential analysis (Table 2) of both field notes and transcripts of the interviews. The purpose of this analysis is to gain an understanding of the meaning of the situations observed by searching for patterns. Using this cultural approach in the analysis allows the researcher to reveal barriers on both an organizational and an individual level. The first author read the data from both the field notes and transcripts of the focus group interviews to obtain an overall impression. The first step of analysis (domain analysis) focused on identifying domains which characterized the situations observed in the field study and discussed in the focus group interviews. This was described by included terms and cover terms. Cover terms refer to a category of cultural knowledge, whereas included terms are the folk terms or situations that are included in the cultural category. Line-by-line coding of words and situations from the data material led to this first analysis. The analysis led to an initial understanding of patterns and meaning of the experiences with EOL conversations. The next step (taxonomic analysis) used these included terms and cover terms from the domain analysis in order to identify the relationship between them and gain a better understanding of the meaning included in the domains. QSR NVivo 10 software (QSR International Pty Ltd., Cardigan, UK) was used to further systematize the domains. The componential analysis formed the basis for the cultural categories, as presented in the results section of this paper, by choosing between the domains in order to answer the research question. Cultural categories refer to components of meaning, which were identified in order to answer the research question. In order to move from the taxonomic to the componential analysis, decisions had to be made. As the data material covered many different domains the cultural categories were chosen to reflect the meaning of the situations and experiences. This was done in collaboration between all three authors.

**Table 2:** Ethnographic analysis (adapted from Spradley 1979, 1980)

Analysis	Content	Example
Domain	The semantic relationship between cover terms and included terms were identified by line-by-line coding of quotes, situations and reflections.	**Included terms** 4-bedroom, office, door open, curtains
		**Semantic relationship** is a kind of
		**Cover term** Environment
Taxonomic	Domains from domain analysis were further systematized using QSR NVivo version 10 software.	**Cover term** Environment
		**Systematized included terms** 4-bedroom and door open = no privacy Curtains = to establish privacy Office = other places to hold a conversation
Componential	The systematized domains were condensed into cultural categories.	**Categories** 1. Physical and organizational setting 2. Timing of, and competencies and roles in, EOL conversation 3. Topics addressed in EOL conversations

### Ethical considerations

The ethical principles of the Declaration of Helsinki [37] were followed in such a way that all respondents were volunteers, were fully informed about the project and were guaranteed anonymity and confidentiality in any subsequent publication. For that reason, the specific hospital, departments and respondent have been anonymized.

Registration and permission from the Danish Data Protection Agency were obtained prior to the study (REG-163-2017).

Research in the field of PC (including EOL conversations) can be characterized as being of a sensitive nature, since both the patient and the HP may be in a vulnerable situation [38]. Therefore, there were several ethical considerations in this study, as follows. During the field study, when following the daily work in the clinic, it was important to establish trust between the HPs and the researcher. This was in order to avoid the HPs feeling judged. For example, on a number of occasions, an HP would ask if the researcher was there to check whether the work was being done correctly. To overcome this the researcher always explained the aim of the study and asked for permission to follow the HP. None of the HPs refused to participate.

When participating in planned conversations with patients and relatives, oral consent was obtained in advance. Since the patients and relatives did not know that the conversation might involve EOL issues, the researcher explained that she was exploring communication about life-threatening diseases between patients and HPs – without mentioning EOL or death, since it was considered unethical to disclose this information. The informal (unplanned) conversations were always conducted after the researcher participated in a planned conversation, so the patient and the relatives were aware of the study.

## Results

The analysis of both the field study and the focus group interviews resulted in three overall categories, which related to: 1. The physical and organizational setting; 2. The timing of EOL conversations and competencies and roles in addressing EOL issues and 3. Topics addressed in EOL conversations.

### “We don’t have time for these things” – the physical and organizational setting

The first category that emerged referred to both the physical as well as the organizational environment in the hospital, which signaled a lack of time and a poor physical environment when EOL issues were addressed.

#### Organizational challenge

During the field study, it was seen on both wards that most of the EOL issues had to be addressed during the daily rounds with very limited time and a lack of privacy. It was emphasized that the daily rounds were a preferred time for having such conversations since there was more medical staff on duty and a senior consultant was present. However, it was not apparent that the senior consultant always took responsibility for these conversations. The daily responsibility for the individual patients was organized in such a way that an interdisciplinary meeting in the morning allocated the patients to the different medical doctors and nurses, with no regard for any possible need for EOL conversations. This meant that inexperienced doctors also had to initiate these conversations if they were given responsibility for a patient. The need for an EOL conversation was expressed by the HP as if the patient was deteriorating and had not been prescribed a level of treatment. If the same doctor was on duty for several consecutive days, efforts were made to ensure that he/she would have the same patients each day. The organizational challenge, however, was that different doctors and nurses were often on duty during the daytime, which may have led to patients having to have the conversation with an unfamiliar doctor or nurse. EOL issues were also addressed at other times than during the rounds – in the focus group interviews nurses mentioned that unplanned EOL conversations occurred, for example when they were alone with the patient in care situations:

*… during personal care, because I’m doing something and the patient doesn’t need to do anything, but it’s as if it gets the patient to open up, and then the conversation is also natural. (Respondent 12)*

*When you’re in the private sphere, which you’re in when you’re having a bed bath, where all the barriers are down, because the nappy has been removed, and, well, so I thought, we’re mentally exposed, but also literally, if you see what I mean. And then the dialogue just happens. (Respondent 2)*

The nurses expressed discomfort with planned formal conversations and preferred the unplanned situations. Spontaneous conversations with a patient having a bed bath, thus being both physically and mentally exposed, might reflect the nurses’ own vulnerability. The nurses’ engagement in these unplanned situations was very different from the doctors’ approach. The doctors needed time to familiarize themselves with the patient’s disease trajectory and preferred to have time for preparation before addressing EOL issues:

*For me, I need to be very familiar with their story … and if they have a really long story, I have to be familiar with that, I mustn’t just sit and faff around with a patient like that. (Respondent 8)*

The expression of “faff around” might refer to a professional attitude in which the doctor sees him/herself as the person who takes on the responsibility for controlling the treatment trajectory and the conversation as well. On the other hand, this attitude might also reflect the doctors’ vulnerable situation – talking to patients about their wishes and hopes, when the patients are faced with an uncertain future.

From both the nurses’ and doctors’ perspectives, a lack of time for EOL conversations was a problem related to the clinical setting. They said that there was not enough time for these conversations, which led to frustrations for the HPs, who expressed a desire to spend time with the patient:

*… but that’s the problem with the current health service, it’s that we don’t have time for these things. There isn’t always time to sit down, either, on a busy night shift. (Respondent 7)*

The respondents also found it frustrating that they had insufficient time for discussions and supervision by their colleagues. Some found ways to talk to their closest colleagues in a private setting, but discussions and supervision regarding EOL conversations were not a formalized part of the working day. This may have consequences for the professional development of the individual HPs when not being able to address their own vulnerability and uncertainty.

When the EOL conversations took place during the ward rounds, the placement and acting in the room signalized the limitation of time. In almost all the EOL conversations observed at the patient’s bedside, the doctor was standing at the front beside the patient (who was in bed or in a chair) and the nurse (if present) was standing in the background.

#### Physical challenges

In both wards, the physical environment was not optimal for EOL conversations. Often the patient would be in a room with one to three other patients (and sometimes their relatives), the beds separated only by a curtain. It was observed that most of the conversations were held in the presence of other patients and this caused many disturbances:

*In the middle of the ward round the door opens and a nurse and porter come barging in. Another patient in the ward has to be moved to another department. They’re raising their voices and they don’t give ANY consideration to the fact that there’s a conversation going on. (Field notes Surgical ward)*

The physical environment and difficulties in obtaining privacy challenged the EOL conversations in such a way that both doctors and nurses expressed frustration regarding this, as it created ethical challenges:

*It’s completely unethical to have to talk to people about such things while there are other people listening in, it’s simply not good enough. (Respondent 5)*

The disruptions also included the door being opened, or the doctor’s phone ringing, which signaled that other things were going on while the conversation took place.

No other (private) rooms for conversations were available in the departments. On some occasions, the conversation took place in a nursing office (with other nurses present documenting their care of other patients in electronic medical journals). In these cases, it was the nurse who took the initiative of preparing the room.

The physical and organizational environment was characterized by the fact that the EOL conversation was held on an equal footing with other activities, during a busy clinical working day with no special attention paid to the individual patient’s need for such conversations. This lack of opportunity to prioritize EOL conversations, due to lack of time and lack of appropriate physical space, may have signaled to the patients that the HPs did not have time to hear about their wishes, hopes and needs for their remaining time. Furthermore, it was rarely possible to provide a physical environment suitable for vulnerable topics, as no rooms were available for the EOL conversation.

### “There’s ‘an instinctive feeling’ when you’re with the patient” – timing of EOL conversations and competencies and roles in addressing EOL issues

The second category was about the appropriate time to address EOL issues, by whom these should be addressed and how the HPs experienced their own competencies and roles in EOL conversations.

#### When to address EOL issues

During the field study it was primarily the doctors who had the role of initiating EOL conversations. The timing was partly determined by health status (the progression/acuteness of the disease) and partly by an intuitive attitude referred to as “an instinctive feeling”. Several respondents in the focus group interviews referred to this term to describe when they thought it was the right time to address EOL issues:

*… so it’s a sort of instinctive feeling; you’re with the patient and you’re doing something or other and you get to a certain point where the patient is open to it, and you have the courage to ask. (Respondent 2)*

This quote tells us that both openness on the part of the patient and a component of courage on the part of the HP need to be present, and that the HP needs to be able to recognize the openness of the patient intuitively. Furthermore, this intuitive attitude was primarily based on the personality and personal engagement of the HP:

*It’s an instinctive feeling, I mean you feel your way towards it. I’ve never been told how to do it or anything. It’s learning by doing. (Respondent 6)*

It was thus required that the individual HP had the ability to perceive the patient’s “openness” and be ready to respond to this openness by asking about the patient’s EOL issues. There was an actual resistance to systematizing EOL conversations. The HPs expressed that they were afraid of offending the patient if they had to formulate and systematize such conversations. The intuitive sense was again brought up regarding the timing of EOL conversations, and the HPs expressed the importance of treading carefully when initiating them:

*I don’t think you can standardise them. You can easily end up treading on people’s toes, so you really have to be sure about etiquette and the actual situation. (Respondent 6)*

The fear of initiating EOL conversations was specifically expressed during the focus group interviews and was considered to be an honest self-awareness:

*I think the [discomfort] I’ve had about it, especially if it’s younger (patients), if the situation is totally clear and we can’t do any more for the person, then there’s no problem with it, but I think it’s hard to set a limit, I mean when it’s time to talk about it, I think that’s hard. It’s hard for me. (Respondent 6)*

The resistance to systematizing the EOL conversations might, therefore, be rooted in the HP’s own uncertainty in a vulnerable situation. This attitude might, however, have consequences for the patients’ need to discuss EOL issues, if it depends on the individual HP’s instinctive feelings about when the timing is right. Furthermore, during the field study, it was seen that the initiation of EOL conversations also depended on how acute the patient’s condition was. It was more likely that the doctor would address EOL issues if the patient was deteriorating rapidly. The patient with a chronic condition, in a severe and limited habitual terminal state, was not necessarily offered the opportunity to talk about EOL issues, even though it might seem that the timing was appropriate when the patient was in a state to be involved and voice their own wishes and opinions.

The difficulty in the timing of EOL conversations was considered during the focus group interviews to be related to the unpredictability of the prognosis in chronic diseases.

*Also because sometimes they can really be at a very very low level and remain there for a very long time, and sometimes it’s unpredictable. I’ve also experienced … having left them to die, that sounds wrong, but I’ve given them palliative care, and then they’ve carried on living anyway. (Respondent 5)*

This unpredictability might increase uncertainty in the individual HP, who is left alone with their instinctive feelings about when the timing is right. When the EOL conversation was initiated, the respondents experienced that the patients were often already aware of their state and were maybe in a “waiting zone” to have this conversation.

*I often think that they want to hear it, and have some confirmation of what they know deep inside … they can sense it, they can sense that the end is drawing near. (Respondent 10)*

*A few of them already know. A lot of them know perfectly well that it’s going in that direction." (Respondent 7)*

These two quotations reveal that patients might be waiting for someone to talk to about EOL issues, while HPs are also waiting for the patients to appear open enough to talk about them. During the field study, especially in the medical ward, it was observed that there were patients with end-stage chronic diseases every day in the department, who might have been candidates for EOL conversations, just waiting for HPs to find the time and courage to recognize the patients’ openness.

#### Roles and feeling a lack of competence

The doctors expressed experiencing that they lacked competence in EOL conversations. They found it problematic and unethical towards the patients that training in addressing EOL issues was carried out while evolving their clinical conversation skills with patients:

*I mean, you can say it comes with experience … and then you can say that it’s not really fair on the patients that they have to wait for you to get experience in having the difficult conversation. (Respondent 8)*

The nurses and social and health care assistant expressed the same feelings of lack of training, but also lack of knowledge during their education, and they called for more knowledge on the topic:

*You’re nowhere near prepared for it by the nursing school. And these things, yes, you get a lot of experience from being involved in it, and you learn things, but it requires background knowledge. Because you’re dealing with some very fragile souls, so you need to know exactly what you’re doing, you need to know that it’s OK to go there. (Respondent 16)*

This lack of competence and knowledge experienced by the HPs placed them in a difficult and vulnerable position, where on the one hand the HPs found themselves expected and obliged to take on their professional role and initiate the EOL conversations, while on the other hand they felt uncertain about how to do that and how to handle such a situation and conversation.

The roles involved in initiating and conducting the EOL conversations were often described as the doctors. In the focus group interviews, all of the HPs said that they saw the doctor as the main character in initiating the conversation, whereas the role of the nurse was less specific and was seen as a kind of “pick up on things” afterwards:

*But it’s the doctor’s job. Perhaps the nurses can come in afterwards and give comfort … and tidy up. (Respondent 10)*

The nurses and health care assistants were hesitant to begin these conversations as they were afraid they would not be able to answer the patient’s questions and did not know how to cope with the reaction the patient might have:

*You run the risk of opening a box, which you won’t be able to figure out how to close again, and it’s extremely dangerous. (Respondent 16)*

In other cases, they would withdraw from answering the patient’s questions in order to protect themselves: they would refer the patient to the doctor and disclaim their own responsibility, even though they sometimes felt that the doctor was unable to conduct the conversation, leaving the patient with unanswered questions, problems and needs:

*I can’t answer that, I can’t comment on that or you’ll have to discuss that with the doctor, but the doctor won’t talk about it, so it’s a bit tricky. (Respondent 3)*

However, the actual care planning (when there was no treatment involved) was seen by some doctors as being a nurse’s job:

*The patient is 100 years old and doesn’t want any more investigation or treatment. At the morning meeting the doctor says that she’s a palliative care patient and the focus should be on fluids, sedatives and painkillers. The patient has had a feeding tube but she rips it out. The question now is what should happen to her – where should she die? The doctor says that the nurse has to figure that out – he doesn’t think there’s anything for the doctor’s rounds today. (Field notes Surgical ward)*

The nurse’s role in addressing EOL issues seemed unclear. As described in the first category, the EOL conversations often occurred spontaneously when the patient was receiving personal care and it felt natural to the nurse to talk about EOL. However, when the conversation was planned and formal a reluctance was expressed. Furthermore, the doctor saw this planning as the role of the nurse. All in all, the HPs did not discuss and clarify roles and responsibilities, which meant that the selection of patients who would be offered EOL conversations was somewhat haphazard.

### “Do you want to be resuscitated?” – topics addressed in EOL conversations

The third category referred to which topics were usually addressed in conversations about EOL matters.

Both during the field study and in the focus group interviews the topics of the EOL conversations revolved around a here-and-now status of the patient’s disease progression and decisions about the level of treatment:

*I think it’s more about what they [the patients] can do right here and now, not what they can expect (in the future), I don’t think it’s that, it’s just: this is how it looks today, and there, that’s where we can do it. (Respondent 2)*

This meant that the conversation centered around what was going on at that point in time and what the plan was for the following day, instead of long-term planning around the patient’s wishes, values and hopes. The consequences of this here-and-now approach might be that the patient was not invited into a shared decision-making process to make plans for the remainder of their life. Furthermore, the topics in the EOL conversations were determined both by the actual status of the patient, as well as by deciding on what not to do, such as no resuscitation (do-not-resuscitate (DNR)) orders and no transfer to the intensive care unit. It varied from doctor to doctor, whether this was a question to the patient and family or merely a statement:

*“Do you want to be resuscitated?” the doctor asks. “I don’t know,” says the patient, crying and angry. The doctor tries to explain to the patient that it will be hard for her to be resuscitated and the nurse agrees and stands there nodding in the background. (Field notes Pulmonary medical ward)*

The doctors claimed that there was an ambiguity in being expected to include the patient in decision-making about resuscitation, knowing that the patient did not really have a choice. In the example above, this ambiguity was demonstrated in the doctor’s and nurse’s attitude and body language, which might have affected the patient in such a way that the latter felt powerless and therefore reacted with anger. The ambiguity was also expressed as problematic:

*That’s why it’s also a problem just to ask, what do you want, to ask the patient, because if the patient doesn’t actually have a choice anyway. (Respondent 6)*

The topics in the EOL conversations were also aimed at talking about the actual dying process, but only if the patient or relatives addressed this. This was a paradox as it was observed that both the patients and their relatives had questions about how the actual dying process would be: whether they would be in pain and whether it would be possible to stay in the hospital during that time.

*They are afraid of getting into the situation where they’re lying there in pain, and they can’t do anything about it themselves, or they feel that they can’t breathe and feel like they’re suffocating. (Respondent 1)*

The HPs ensured the patients and relatives that they would do whatever they could to relieve their symptoms when the time came.

*“Your pain is going to be managed and it’s done according to the level of pain, where you will get morphine, arthritis medication and Paracetamol. But you must remember to tell us when you’re in pain, so we know how much you need” the doctor said. (Field notes Surgical ward)*

As seen in this quote, the doctor is involving the patient in decisions about pain management and treatment in order to make the patient feel safe. However, the quote also illustrates a biomedical approach to “problem-solving”: if the patient experiences pain, then they will be given analgesics. The patient’s existential pain and suffering are thus not addressed by the HP but are left to the patient to contemplate alone.

The EOL conversation was also viewed as a process by the respondents in the interviews. It was not restricted to one single conversation, as not all topics could be covered in one conversation. Rather, there was a series of conversations, which addressed the current status of the disease each day. However, this issue was ambiguous, since the doctors did not always have enough time for more than one conversation:

*The nurse tells the doctor that the patient’s son or daughter would like to talk to them. They had a long conversation yesterday and the doctor says that they can’t have a conversation for half an hour every single day. There just isn’t time for that. (Field notes Pulmonary medical ward)*

The lack of time, as also described in the first category, might mean that the topics the patient and relatives wish to address in EOL conversations cannot always be accommodated and may never be addressed. Additionally, the ‘lack of time’, as described in the field note above, might in fact be attributable to EOL conversations focusing on the deterioration of the patient’s disease status, the level of treatment and what not to do, rather than the longer-term planning of care.

## Discussion

The results of this study provide an insight into how EOL issues are addressed in an acute care hospital setting. As described in the background section, for a number of years PC in hospital has been described as an unsuitable setting for patients suffering from life-threatening illnesses and nearing the end of their lives. This study confirms that this is still the case, as many challenges are present when addressing EOL issues.

Firstly, both the physical and organizational environment were identified as presenting barriers for talking about EOL matters. As most of the conversations had to take place on the daily ward rounds, there was limited time and space for EOL matters. The lack of privacy in a hospital setting was described by the HPs as particularly “unethical”; it created a dilemma since they sometimes had to engage in EOL conversations in the presence of others. In this study, the EOL conversations took place in both a single room (for just one patient) and in rooms with up to four patients, which meant that three other patients could be present at the time when the EOL conversations took place, with only a curtain separating the beds. Some HPs made the effort to secure a quiet, private room, but there was no consistency in this matter and often no such rooms were available. Previous research has found the environmental setting in the hospital to be challenging [39, 40], as the lack of privacy and the noise in an acute care ward do not allow the patients and relatives to have a calm and aesthetic room at the end of life. EOL conversations cover sensitive information, which may call for a quiet and private environment. The fact that the environmental frames were inhibiting caused ethical dilemmas for the HPs, who felt that they were unable to live up to what they valued as important elements in EOL care.

Nurses experienced that the patients often initiated EOL at a time when a private space had been created. The nurses described this as being spontaneous, indicating that EOL issues are not only discussed during the planned time on daily ward rounds, but are a constant matter of concern for the patients. The creation of a private intimate room was also important for the nurses. The feeling of reluctance toward the planned conversations might be explained by the vulnerability that nurses (and doctors) might feel in engaging in these conversations. Courage and vulnerability in nursing have been described by Thorup et al. [41], who argue that vulnerability and suffering shape the nurses’ courage and give them the ability to help the patients in facing their own suffering and vulnerability. Creating that private space in an acute care setting might be encouraging for the patient, as well as giving the nurses the courage to talk about EOL issues.

The organizational challenge in this study was also related to the workflow, e.g. how the patients were allocated to the different nurses and doctors. It was said that the senior consultant ought to have significant involvement in EOL conversations and therefore preferably address such matters during the daily ward rounds. Earlier research has shown that the organizational barriers leave it up to the individual HP to organize the daily care and treatment, creating fragmented care without continuity [42], which also seems to be a consequence for the EOL conversations in this study. Furthermore, it was also claimed that there was a lack of time to conduct EOL conversations – this has been described as a well-known barrier to conducting PC in a hospital setting [26, 43–45]. However, this study contributes to the knowledge that EOL conversations take place alongside other activities on daily ward rounds and that no extra time or focus are given to them.

Secondly, this study finds that the initiation of EOL conversations depends on the readiness, not only of the patient, which has been thoroughly described in previous studies [46–48], but also of the individual HP, who often initiates the conversations intuitively. The question of why there is no systematic implementation of conversation initiatives, like the ACP, might be answered partly by this intuitive initiation of EOL conversations. There is a risk that the intuitive sense in the individual HP may not match the readiness of the patient and their relatives. The notion of courage may relate both to interacting with the patient on sensitive matters and also to relate to one’s own anxiety about death, which may be a factor regarding reluctance to addressing EOL matters and caring for the dying [49, 50]. In this study, it was merely expressed as discomfort and unease regarding when to draw the line and knowing when the time was right to stop treatment. The consequence may be that personal factors like the doctors’ discomfort and lack of courage may be inhibiting them in addressing EOL topics. Combined with the fact that in Denmark there are no formal guidelines regarding EOL conversations, this might lead to a delay or maybe even to no conversation at all.

The HPs felt that the patients were waiting for them to address EOL matters. This corresponds with previous research by Barnes et al. [51], who found that the majority of patients with progressive cancer thought that it was the doctor’s responsibility to initiate EOL discussions, although the patients felt that the doctor seemed reluctant to do this. There seems to be a crucial point at which both the patient and the HP are ready to address EOL matters. If that point is missed there is a chance that the patient will not be given the opportunity to express their wishes and thoughts on EOL matters. It was observed in this study that the initiation also depended on the disease status of the patient. If they were in a state of acute deterioration, then the doctors “had to” address EOL matters (especially level of treatment) and then both needed “to be ready”. Competencies and training were viewed as important in order to address this readiness. Previous research [19, 20] shows that there is a profound lack of training among nurses and doctors in EOL conversations, which may lead to an actual avoidance of initiating EOL matters [19], which is supported by the results in this study. It was seen that the doctors and nurses felt that EOL conversations should be initiated by the doctor. In Denmark, the law states that only doctors have the authority to decide on and document the level of treatment for the patient [52]. However, the nurses’ role in EOL conversations was unclear, and was described by the doctors as “picking up on things afterward”. This supports previous findings, which indicate that although nurses may feel competent to take responsibility for the difficult conversation, they are often hesitant as they consider this task to be the doctor’s responsibility [53]. In a study by Mehta et al. [54] nurses express that they felt competent to conduct EOL conversations independently [54]. However, in our study, the nurses and social and health care assistant expressed reluctance and fear of saying or doing something wrong to the patient. It was also apparent that the definition of “EOL conversation” was not always clear. For the doctors, it was often perceived as the conversation where the level of treatment was discussed, but for nurses it appeared also to mean a conversation that occurred spontaneously and contained other sensitive matters regarding EOL. The clarification of the roles should therefore also be related to the different aspects that EOL conversations may contain.

Thirdly, the topics of the EOL conversations were shown in this study to be dominated by what could be called “decisions of omission” (do-not-resuscitate and do not admit to the intensive care unit) and how to relieve symptoms in the actual dying process. The discussion on thoughts and wishes from the patients and their relatives about the patient’s remaining life was addressed only to a lesser extent. Even though the HP was obliged to engage patients and relatives in this decision-making process, the doctors claimed that the patients did not always have a choice in making these decisions (as there may be clinical difficulties regarding resuscitation). This created an ethical challenge and frustrations in the doctors, as they felt they were providing false hope for the patients. It has been described earlier that reluctance towards do-not-resuscitate decision making may be related to a misunderstanding among the HPs that do-not-resuscitate also means withholding other life-sustaining activities, such as giving blood or antibiotics [55]. A recent Swedish study by Pettersson et al. [56] found that only half of the HPs in a hematology and oncology department found it likely that the patients would be involved in decision making on do-not-resuscitate, even though they felt that it was important to involve them. The discussion on whether hospital is the ideal place for discussing do-not-resuscitate and EOL matters has been investigated by Robinson et al. [57], who reported that 86% of patients in primary care actually preferred to discuss do-not-resuscitate matters with their own family physician and 56% felt that this should happen while they were in a healthy state. This indicates that EOL matters ought to be addressed in primary health care as well as outpatient clinics. However, it was a fact that these matters had not been addressed until the patients were hospitalized (and often very late in the disease trajectory), making hospital HPs important actors in EOL conversations.

In order to move forward in addressing EOL matters in a hospital setting, it may be of importance to take into account the fact that PC is, by nature, an interdisciplinary approach [58]. This study illustrates that roles are poorly defined and calls therefore for clarification, owing to the fact that EOL matters are addressed not only on the daily ward rounds but also when the nurses are alone with the patients. This makes the EOL conversation a process, rather than a single conversation to be undertaken on the daily ward rounds.

### Rigor and limitations

Throughout the study methodological rigor was attained by using the qualitative concepts of relevance, validity, and reflexivity, as described by Malterud [59]. The relevance of the study was ensured partly by a thorough initial literature search and partly by developing the research question in collaboration with HPs and participants from the board of patients and relatives in the hospital. The results were also presented to the members and discussed, allowing them to object if they did not recognize the hospital practice from their perspective as patients and HPs. Furthermore, everyone asked agreed to participate in the field study, and all were supportive that the topic should be explored.

Reflexivity was ensured by discussion between the authors, both during the data collection phase and in the analysis. Conducting participant observation carries the risk of affecting what is taking place in daily practice. The fact that the first author was a registered nurse and wore a uniform during her observations made it possible for her to “blend in” with the rest of the staff and avoid drawing too much attention to herself. Furthermore, the first author kept a reflective diary during the field study, discussing issues such as staff reluctance towards the study being carried out. Validity was ensured by presenting the quotes and field notes as transparently as possible in the results section in this paper. Furthermore, the study used the COREQ-criteria for the reporting of qualitative research.

The study also had limitations. It took place in two wards of a single hospital in Denmark, which may be perceived as a small sample. However, using ID as the design allows the study to be focused and not necessarily engage in prolonged field studies, since ID strives merely to generate knowledge.

The focus on EOL conversations at the generalist PC level in an acute care setting was difficult since patients were not necessarily identified as having PC needs. This was acknowledged beforehand. The HPs and the researcher therefore collaborated to identify relevant EOL conversations in which the researcher could participate. However, the HPs in the study appeared to have different perceptions of what an EOL conversation is. For the doctors, it was a conversation regarding the level of treatment, whereas for the nurses it encompassed a number of EOL topics. Thus there may have been EOL conversations taking place without the knowledge of the researcher. The fact that the conversations were primarily observed during the daytime may also have inhibited the researcher from becoming fully acquainted with the EOL conversation in all its variations.

### Implications for practice

Since this study followed an ID design it is important to reflect upon how the results can have an impact on clinical practice and contribute to action.

The lack of overall guidelines and strategies on how EOL conversations should take place in a hospital setting means that the individual wards and HPs are left to decide how they manage the task. In order to create a culture where the EOL conversations in hospital address not only the level of treatment but also the patient’s wishes regarding the remainder of their life, several points must be clarified and discussed.

Clarification of how to address EOL conversations systematically, so that they are not merely a matter of the doctor’s or nurse’s intuition, is both a national and local responsibility. Local policies must be developed to ensure that sufficient time is assigned to EOL conversations, that private rooms are made available and that the conversations address both the level of treatment and the patient’s wishes for their remaining life.

Furthermore, the planning of policy regarding EOL conversations requires a focus on the necessary HP competencies, as well as organizational changes, in order to make time and space available for such conversations to take place. As care workers spend more time with their patients, and EOL conversations sometimes occur without the doctor’s involvement, the policy must address the rules and roles for each HP in addressing EOL issues. The interdisciplinary approach should ensure that EOL conversations form part of a process involving several actors, rather than depending on a single doctor. Training is needed to strengthen conversation skills and to help HPs work with their own vulnerability in talking about EOL. As clinical decision-making requires both a professional and a systematic approach, combined with intuitive understanding, both must be taken into account in future policies and educational strategies. This could be addressed in basic nursing and medical education, and also as an additional training program for experienced HPs. The results of this study indicate that an interdisciplinary training course would be appropriate for clarifying roles.

As EOL conversations will always be individual and cannot be standardized to apply to all patients, further research must focus on developing tools that can be systematically implemented at a national level, and which can also work for the individual patient.

## Conclusion

There are a number of barriers that need to be overcome in order to talk about EOL issues with patients suffering from life-threatening illnesses in an acute care hospital setting, not least the physical and organizational environment. In order to provide patients and their relatives with better opportunities to express their EOL wishes, there is a need for an interdisciplinary approach to EOL conversations with clearer roles and guidelines. There is also a need for improved staff competencies and changes to the organizational and physical environment.

## Data Availability

The datasets analysed during this study are available from the corresponding author on reasonable request.

## Abbreviations

EOL: End of life
HP: Healthcare professional (can refer to a doctor, nurse or social and healthcare assistant)
ID: Interpretive Description
PC: Palliative Care

## Footnotes

1 In Denmark palliative care (PC) is organized on two levels: generalist PC and specialist PC. Generalist PC is performed by any healthcare professionals who are not part of a specialist palliative care team. This can include hospitals and primary care.
